# Supplementary figures and images for: IL-6 trans-signaling mediates cytokine secretion and barrier dysfunction in hantavirus-infected cells and correlates to severity in HFRS
Source: PLoS Pathog. 2025 Apr 9;21(4):e1013042. doi: 10.1371/journal.ppat.1013042 (PMC12054857; doi:10.1371/journal.ppat.1013042)

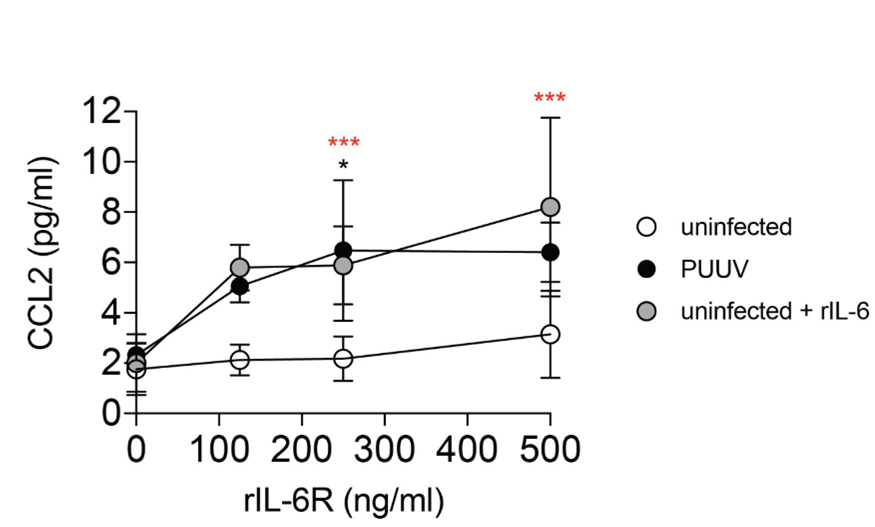

Supplement: S1 Fig — Levels of CCL2 in supernatants of uninfected and infected HUVECs treated with sIL-6R and/or rIL-6, or left untreated (n=3, except n=2 for 125 ng/ml). Symbols depict mean and error bars indicate SD. Two-way ANOVA followed by Dunnet’s or Šídák’s multiple comparison test. Black asterisks indicate significance when comparing PUUV to uninfected. Red asterisks indicate significance when comparing each sIL-6R-treated conditions of PUUV-infected cells with untreated PUUV-infected cells. *, p<0.05; **, p<0.01; ***, p<0.001, ****, p<0.0001. (TIF) [file ppat.1013042.s001.tif]

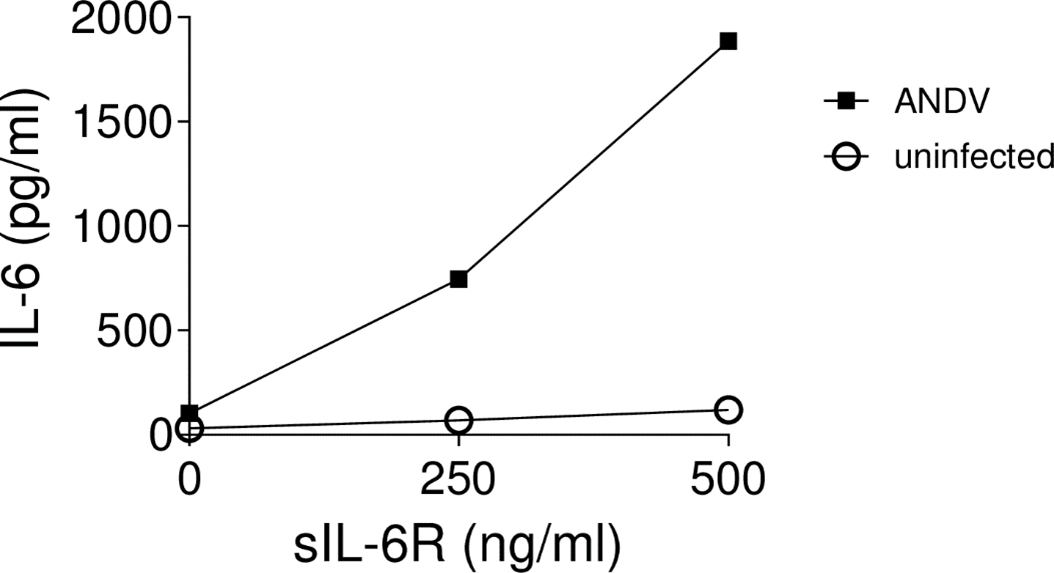

Supplement: S2 Fig — Levels of IL-6 in supernatants of ANDV-infected and uninfected HUVECs treated with sIL-6R (250 or 500 ng/ml) or left untreated (n=2; data shown represent the mean value). (TIF) [file ppat.1013042.s002.tif]

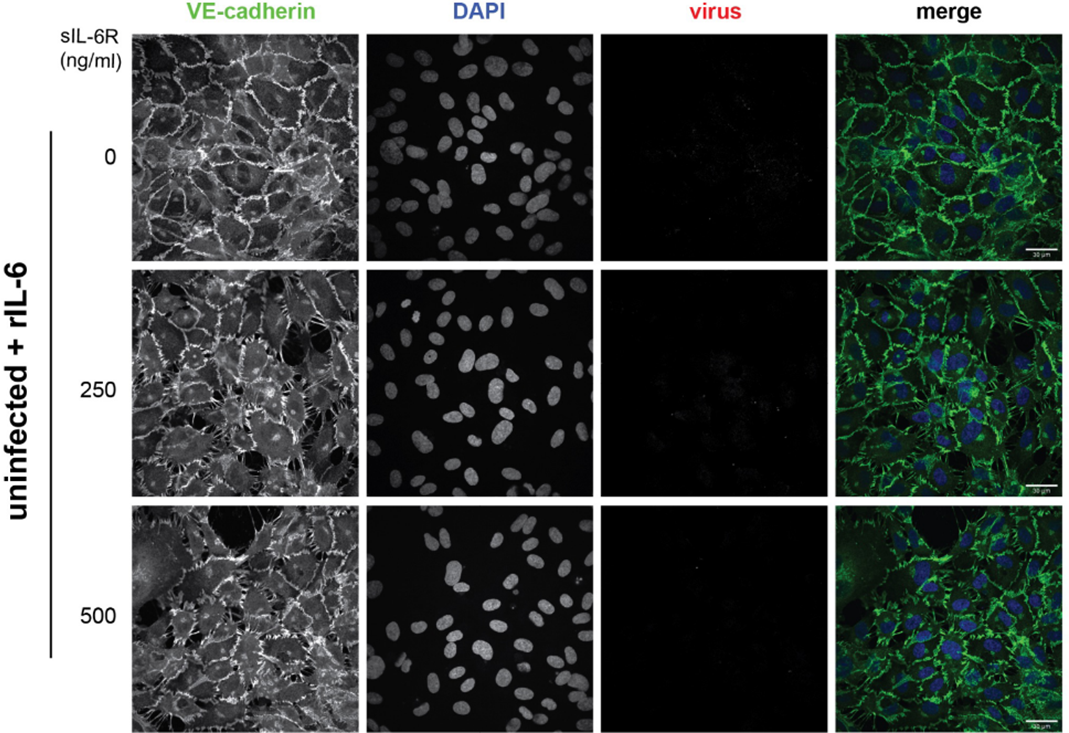

Supplement: S3 Fig — Immunofluorescence images showing expression of DAPI (blue), virus (red), and VE-cadherin (green). Representative images of three independent experiments are shown. (TIF) [file ppat.1013042.s003.tif]

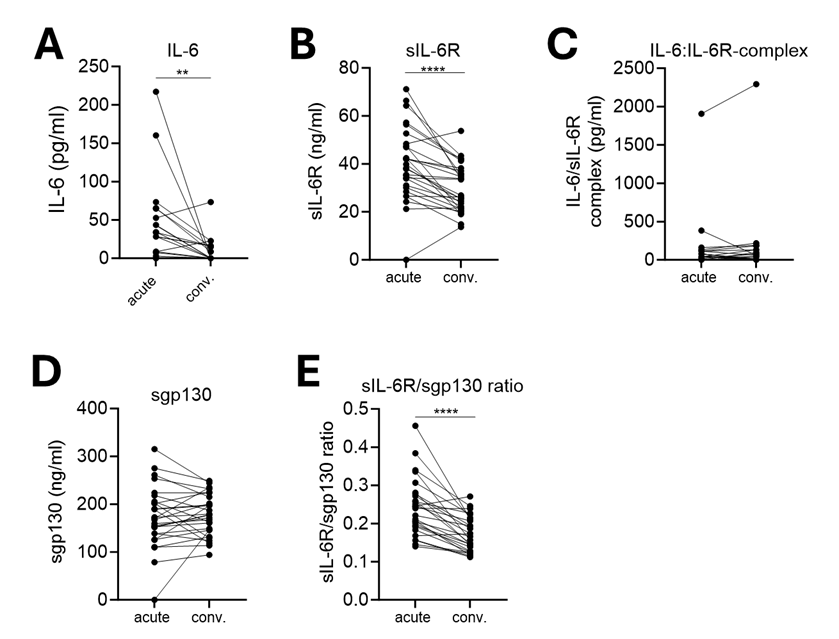

Supplement: S4 Fig — Plasma levels of (A) IL-6, (B) sIL-6R, (C) IL-6:sIL-6R complex, and (D) sgp130 in acute and convalescent HFRS patients (n=28). (E) Ratio of plasma sIL-6R and sgp130 in HFRS patients (n=27). Wilcoxon signed-rank test. **, p<0.01; ****, p<0.0001. (TIF) [file ppat.1013042.s004.tif]
